# Identification of mRNAs related to endometrium function regulated by lncRNA CD36–005 in rat endometrial stromal cells

**DOI:** 10.1186/s12958-018-0412-4

**Published:** 2018-10-15

**Authors:** Xueying Zhang, Ying Xu, Lulu Fu, Dandan Li, Xiaowei Dai, Lianlian Liu, Jingshun Zhang, Lianwen Zheng, Manhua Cui

**Affiliations:** 1 Reproductive Medical Center, Department of Obstetrics and Gynecology, The Second Hospital of Jilin University, No. 218 Ziqiang Street, Changchun, 130041 Jilin China; 2 Department of Obstetrics and Gynecology, The Second Hospital of Jilin University, No. 218 Ziqiang Street, Changchun, 130041 Jilin China

**Keywords:** lncRNA, CD36–005, RNA sequencing, Endometrium, Stromal cells, PCOS

## Abstract

**Background:**

Polycystic ovary syndrome (PCOS) is a heterogeneous endocrine disorder in women of reproductive age and is commonly complicated by adverse endometrial outcomes. Long non-coding RNAs (lncRNAs) are a class of non-protein-coding transcripts that are more than 200 nucleotides in length. Accumulating evidence indicates that lncRNAs are involved in the development of various human diseases. Among these lncRNAs, lncRNA CD36–005 (CD36–005) is indicated to be associated with the pathogenesis of PCOS. However, the mechanisms of action of CD36–005 have not yet been elucidated.

**Methods:**

This study determined the CD36–005 expression level in the uteri of PCOS rat model and its effect on the proliferation activity of rat primary endometrial stromal cells. RNA sequencing (RNA-seq) and bioinformatics analysis were performed to detect the mRNA expression profiles and the biological pathways in which these differentially expressed mRNAs involved, after CD36–005 overexpression in the primary endometrial stromal cells. The differential expression of Hmgn5, Nr5a2, Dll4, Entpd1, Fam50a, and Brms1 were further validated by quantitative reverse transcription polymerase chain reaction (qRT-PCR).

**Results:**

CD36–005 is highly expressed in the uteri of PCOS rat model and promotes the proliferation of rat primary endometrial stromal cells. A total of fifty-five mRNAs differentially expressed were identified in CD36–005 overexpressed stromal cells. Further analyses identified that these differentially expressed mRNAs participate in many biological processes and are associated with various human diseases. The results of qRT-PCR validation were consistent with the RNA-seq data.

**Conclusions:**

These data provide a list of potential target mRNA genes of CD36–005 in endometrial stromal cells and laid a foundation for further studies on the molecular function and mechanism of CD36–005 in the endometrium.

**Electronic supplementary material:**

The online version of this article (10.1186/s12958-018-0412-4) contains supplementary material, which is available to authorized users.

## Background

Polycystic ovary syndrome (PCOS) is one of the most common and complex endocrine disorders in women of reproductive age, with a prevalence estimated to be 5–10% [1–4]. The clinical features of PCOS are highly heterogeneous. Patients with PCOS have reproductive dysfunction and metabolic abnormalities, and are commonly characterized by persistent ovulatory disorder, ovarian polycystic morphology, hyperandrogenism, insulin resistance (IR), hyperinsulinemia, and obesity [5, 6]. In addition, in women with PCOS, the risk of type 2 diabetes, cardiovascular disease, infertility, and some adverse endometrial outcomes increases [7–11]. The diversity of the clinical features of PCOS is attributed to the multifactorial contribution on its pathogenesis, including complex genetic and environmental factors [12]. Patients with PCOS often have endometrial abnormalities and most are anovulatory or oligo ovulatory. However, after the anovulation or oligo ovulation is treated, they still have lower pregnancy rates and higher spontaneous miscarriage rates, which suggest the decrease of their endometrial receptivity [10, 13]. Additionally, patients with PCOS have a significantly higher risk of having endometrial hyperplasia and developing endometrial cancer [11]. These adverse endometrial outcomes are associated with the metabolic abnormalities of PCOS including chronic unopposed estrogen, IR, hyperinsulinemia, hyperandrogenism, and obesity, and complex genetic alterations [11, 14]. However, the underlying mechanisms of PCOS in the uterus are still unclear.

Long non-coding RNAs (lncRNAs) are defined as a class of non-coding transcripts with the length of more than 200 nucleotides. Although lncRNAs lack the capacity to code for proteins, they can regulate gene expression at epigenetic, transcriptional, posttranscriptional, and other levels [15]. lncRNAs are proven to play key roles in many biological processes, including genetic imprinting, X-chromosome inactivation, gene transcription regulation, organelle biogenesis, and subcellular trafficking [16]. Dysfunctional lncRNAs contribute to the pathogenesis of many human diseases, such as diabetic nephropathy, nonalcoholic steatohepatitis, cardiomyopathy, atherosclerosis, and cancers in various systems [17–21]. The role of lncRNAs in the pathogenesis of several endometrial diseases has also been reported in recent studies, including implantation failure or spontaneous miscarriage, endometrial hyperplasia, adenomyosis, endometriosis, and endometrial cancer [11, 22–24]. However, we knew little about the role of lncRNAs in the pathogenesis of adverse endometrial outcomes of PCOS.

In our previous research, we found that lncRNA CD36–005 (CD36–005) was significantly upregulated in the ovaries of PCOS rat model by lncRNA expression profile analysis [25]. After determining that CD36–005 is also highly expressed in the uteri of PCOS rat model in the present study, we suggest that the upregulation of CD36–005 expression might be associated with the pathogenesis of PCOS in the uterus. We used primary endometrial stromal cells from rat uteri as the in-vitro model, and performed RNA sequencing (RNA-seq) technology and bioinformatics analyses after CD36–005 overexpression to investigate its potential role from a more comprehensive perspective. We also conducted CCK-8 assay to determine the effect of CD36–005 on the proliferation activity of stromal cells, which is the first step of decidualization. Our results provide insights into the underlying molecular mechanisms of CD36–005 in the regulation of endometrial stromal cells and the pathogenesis of adverse endometrial outcomes of PCOS.

## Methods

### Animals

All animal procedures were approved by the Institutional Animal Care and Use Committee of Jilin University, and were conducted in accordance with the Guidelines for the Care and Use of Laboratory Animals. Mature female Wistar rats (8 weeks old) were purchased for the isolation of primary endometrial stromal cells in the present study.

### Animal model, vaginal smears, tissue sampling, and hormone assays

The uterine tissue used in this study was collected from the PCOS rat model in the article “Expression profiles of mRNA and long noncoding RNA in the ovaries of letrozole-induced polycystic ovary syndrome rat model through deep sequencing [25].” The middle of the uteri (body of uterus) was collected for subsequent total RNA extraction.

### Isolation of endometrial stromal cells

Primary endometrial stromal cells were isolated from 8-week-old Wistar rats’ uteri using previously described methods, cultured with DMEM-nutrient mixture F-12 Ham (DMEM-F12, Hyclone, USA) containing 10% heat-inactivated fetal bovine serum (FBS, Gibco, USA), and incubated at 37 °C with 5% CO_2_ [26].

### Cell immunofluorescence

Stromal cells were seeded on coverslips and washed with phosphate buffer saline (PBS, Hyclone, USA). Then, the confluent cells were fixed in cold methanol, permeabilized with 0.1% Triton X-100, and blocked with 1% goat serum. Stromal cells were incubated overnight at 4 °C with rabbit anti-mouse monoclonal antibody specific to vimentin and cytokeratin-19 at a 1:200 dilution in PBS (Boster, Wuhan, China) [27–29]. In order to validate the specificity of vimentin antibody, 5% nonimmune goat serum was used as negative control. After washed by PBS three times, stromal cells were incubated with goat-anti-rabbit second antibody (Boster, Wuhan, China) for 2 h at room temperature. Finally, coverslips were rinsed and mounted with DAPI. The stained stromal cells were observed using Olympus IX71 fluorescence microscope and images were analyzed by using cellSens Dimension.

### Overexpression and knockdown of lncRNA CD35–005 in endometrial stromal cells

When stromal cells reached 70% confluency, they were transiently transfected with either Ad-CD36–005 or Ad-GFP designed by Hanbio Biotechnology Co., Ltd. (Shanghai, China) at the designated multiplicity of infection (MOI) of 50. The full-length sequence of CD36–005 was directly cloned into the pHBAD-EF1-MCS-3flag-CMV-GFP vector by seamless cloning. After 6 h incubation at 37 °C in 5% CO2, the medium was replaced with fresh growth medium. After transfection with Ad-CD36–005 or Ad-GFP, the stromal cells were collected at 48 h.

The small-interfering RNA (siRNA) duplexes for targeting CD36–005, as well as a scrambled sequence (control siRNA duplex, negative control) were synthesized by the RiboBio Company (Guangzhou, China). The sequences were shown as follows: 5’-UAAGGACCUCUAUUGCUUGTT and CAAGCAAUAGAGGUCCUUATT (CD36–005 siRNA); 5’-UUCUCCGAACGUGUCACGUTT and 5’-ACGUGACACGUUCGGAGAATT (nonspecific scrambled siRNA, negative control). Transfections for siRNA were performed according to Fugene HD Transfection Reagent (Promega, USA) protocol. After transfection with CD36–005 or control siRNA, stromal cells were collected at 36 h.

### Cell proliferation

CCK-8 reagent (Promega, USA) was used to perform the proliferation assays according to the manufacturer’s directions. Stromal cells were seeded at a density of 1 × 10^5^ /well in 96-well plates and cultured in the DMEM/F12 medium containing 2% heat-inactivated FBS. After transfection with Ad-CD36–005, Ad-GFP, or CD36–005 siRNA, the stromal cells were cultured for 48 h. Finally, cells in each well were added with 10 μl of CCK-8 reagent and incubated for 2 h. Absorbance was measured at 490 nm using a 96-well plate reader.

### RNA extraction

Total RNA from the middle of the uteri and stromal cells were extracted using TRIzol (Invitrogen/Life Technologies, USA) according to the manufacturer’s protocol. The concentration and quality of RNA were determined using NanoDrop 2000 spectrophotometer (Thermo Fisher Scientific, UK) to ensure that the OD260/280 absorbance ratios of all samples were between 1.8 and 2. RNA integrity was evaluated using the Agilent 2100 Bioanalyzer (Agilent Technologies, USA).

### RNA sequencing and bioinformatics analysis

RNA samples from three stromal cells in each group with RNA Integrity Number (RIN) ≥ 7 were subjected to the subsequent mRNA sequencing by Shanghai OE Biotech Co., Ltd. (Shanghai, China). The libraries were constructed using TruSeq Stranded mRNA LTSample Prep Kit (Illumina, San Diego, CA, USA) according to the manufacturer’s instructions. These libraries were sequenced on the Illumina sequencing platform (HiSeqTM 2500 or Illumina HiSeq X Ten) and 125 bp/150 bp paired-end reads were generated. We used Gene Ontology (GO) to categorize the function of the differentially expressed mRNAs and Kyoto Encyclopedia of Genes and Genomes (KEGG) to predict the signaling pathways in which these differentially expressed mRNAs may be involved.

### Real-time quantitative PCR analysis

Reverse transcription reactions were performed using PrimeScript RT Reagent Kit (Takara Bio) according to the manufacturer’s protocol. Quantitative real-time polymerase chain reaction (qRT-PCR) analyses were performed at the following conditions: 95 °C for 2 min followed by 40 cycles of 95 °C for 15 s and 59 °C for 30 s, according to the instructions of SYBR Premix Ex Taq (TaKaRa). The mRNAs and lncRNA CD36–005 were normalized to glyceraldehyde-3-phosphate dehydrogenase (GAPDH), and the relative expression levels were analyzed by calculating the fold changes using the 2^−ΔΔCt^ value method. We purchased the primer sequences for qRT-PCR from RiboBio Company (Guangzhou, China).

### Statistics

Significance of difference between two groups was compared by Independent-Samples T Test. Data are shown mean ± SEM. Significance of difference was considered significant at *P* < 0.05. All statistical analyses were performed using SPSS17.0 software (SPSS Inc., Chicago).

## Results

### Quality control of primary endometrial stromal cell cultures

**Fig. 1:**
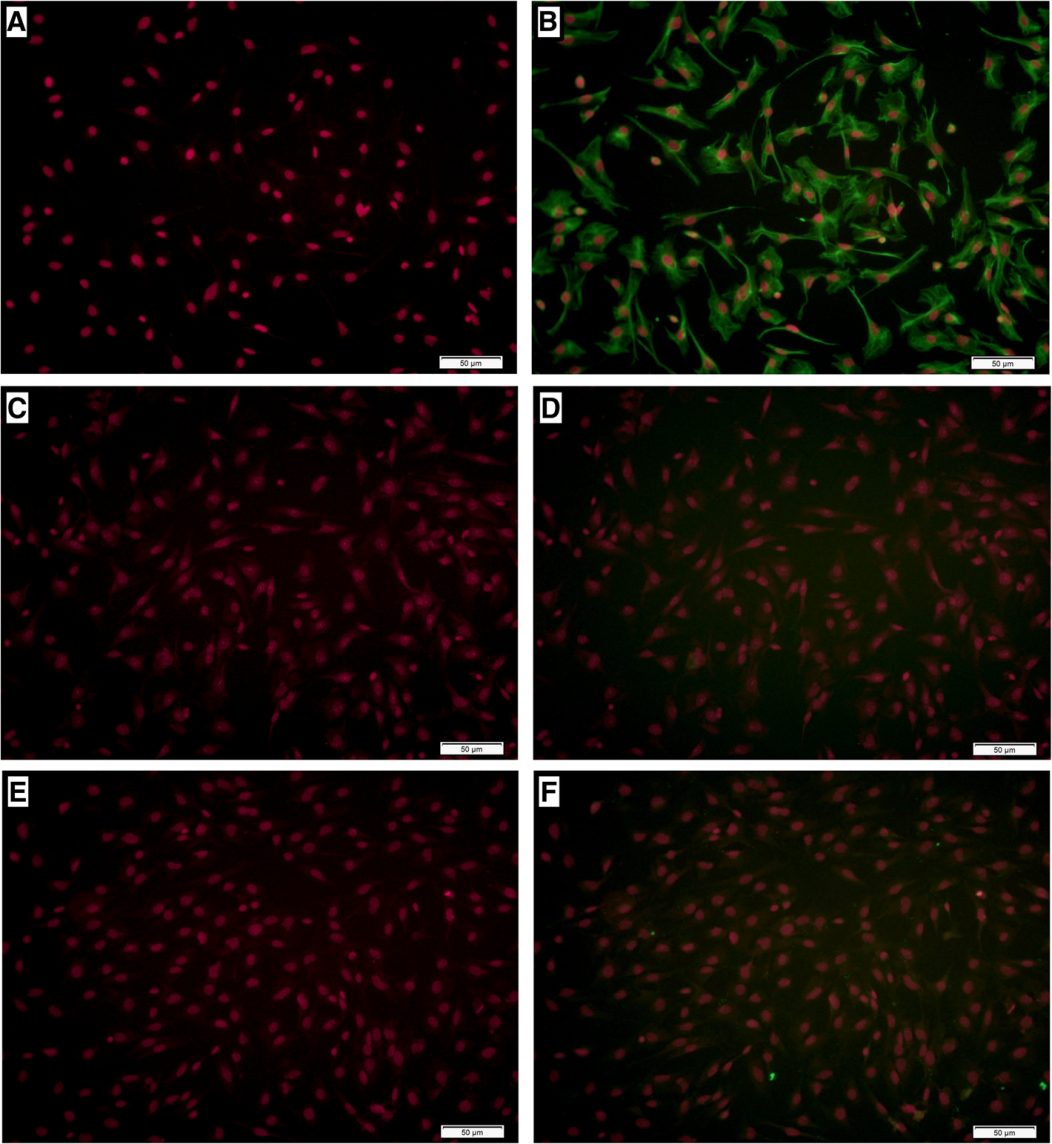
Immunofluorescence for vimentin in endometrial stromal cells. **a, c, e** Only DAPI staining on endometrial stromal cell cultures. **b** Vimentin double staining on endometrial stromal cell cultures. **d** Negative control with primary antibody omitted for endometrial stromal cell cultures. **f** Cytokeratin-19 double staining on endometrial stromal cell cultures. The scale bar is shown in the lower right corner of each picture. The length of the scale bar is equivalent to 50 μm

The purity of the primary endometrial stromal cells was assessed using the difference in vimentin and cytokeratin expression. The absence of primary antibody was used as a negative control. Results of cell immunofluorescence show that the purity of endometrial stromal cells was more than 90%, which can be used for subsequent experiments (Fig. 1).

### Expression of lncRNA CD36–005 in the uteri of PCOS model

**Fig. 2:**
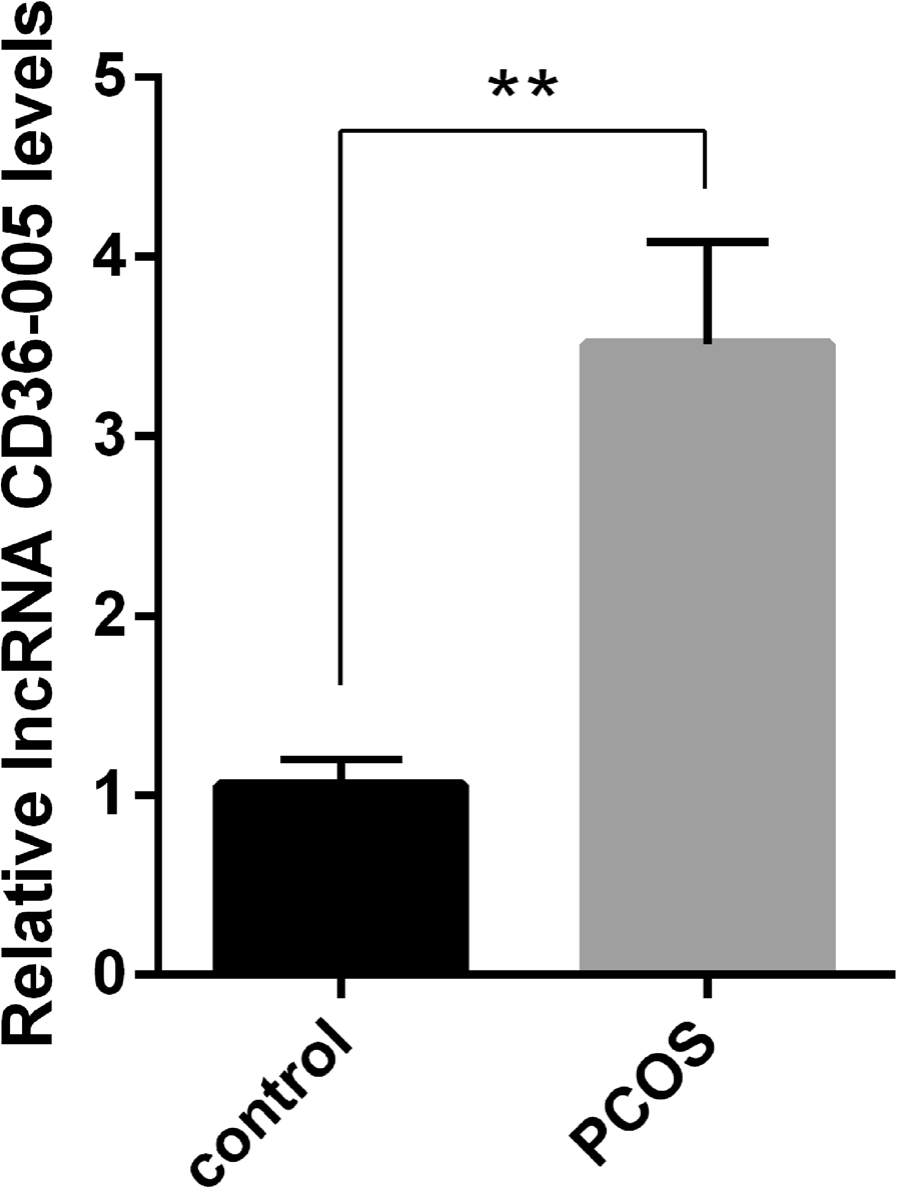
Relative expression of lncRNA CD36–005 was quantified by qRT-PCR. Compared with the control group, CD36–005 displayed significantly increased expression in the uteri from PCOS rat model (* *P* < 0.05; ** *P* < 0.01). Data are shown mean ± SEM

Result of the qRT-PCR analyses showed that the expression level of CD36–005 in the uteri of PCOS rat model was significantly higher than in the normal (Fig. 2).

### Overexpression and knockdown of lncRNA CD36–005 in primary endometrial stromal cells

**Fig. 3:**
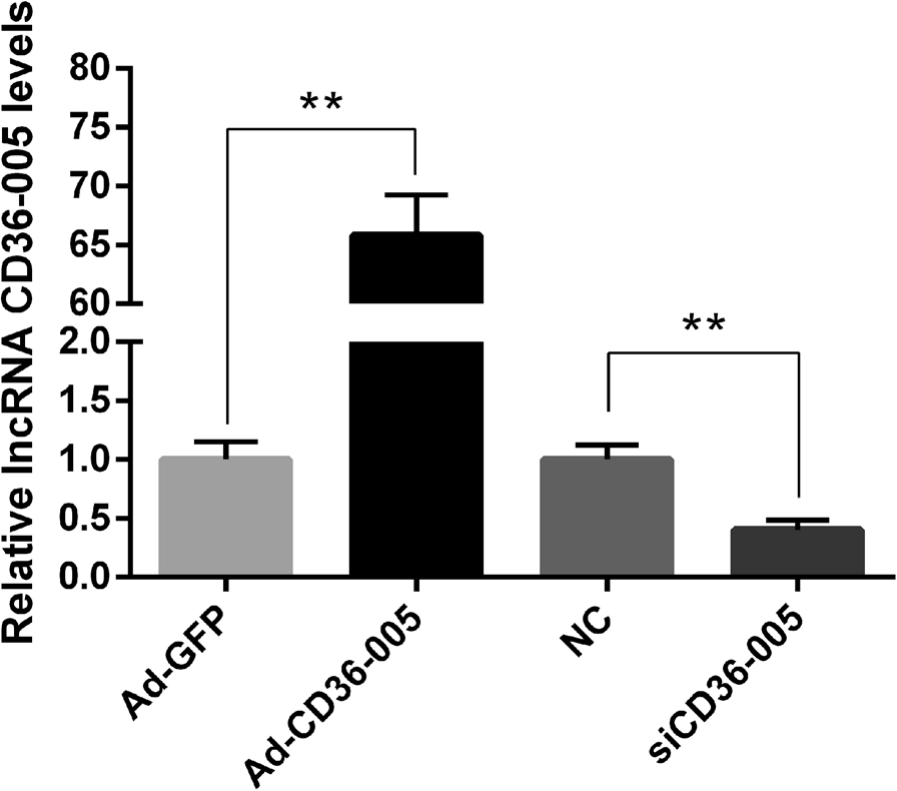
Relative expression of lncRNA CD36–005 in the endometrial stromal cells after transfected with Ad-GFP, Ad-CD36–005, control siRNA and CD36–005 siRNA. The CD36–005 expression was significantly increased after stromal cells were transfected with Ad-CD36–005. The CD36–005 expression was significantly decreased after stromal cells were transfected with CD36–005 siRNA (* *P* < 0.05; ** *P* < 0.01). Data are shown mean ± SEM. The raw data for Fig. 3 were provided in Additonal file 1: Table S1

Result of the qRT-PCR analyses showed that the expression level of CD36–005 in the Ad-CD36–005 transfected stromal cells was significantly higher compared with that of Ad-GFP transfected cells; CD36–005 expression level in the CD36–005 siRNA transfected stromal cells was significantly lower compared with that of the negative control siRNA transfected cells (Fig. 3).

### Effects of lncRNA CD36–005 on primary endometrial stromal cell proliferation

**Fig. 4:**
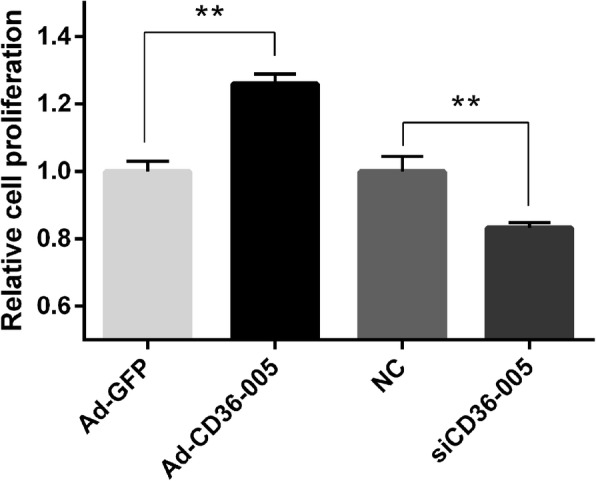
The effect of lncRNA CD36–005 overexpression and knockdown on the proliferation of endometrial stromal cells. The stromal cells proliferation was significantly increased after stromal cells were transfected with Ad-CD36–005. The stromal cells proliferation was decreased after stromal cells were transfected with CD36–005 siRNA (* *P* < 0.05; ** *P* < 0.01). Data are shown mean ± SEM. The raw data for Fig. 4 were provided in Additonal file 2: Table S2

Overexpression of CD36–005 could strengthen the proliferation activity of stromal cells. On the contrary, the proliferation activity of stromal cells was reduced compared with control after they were transfected with CD36–005 siRNA (Fig. 4).

### Gene expression profiling following CD36–005 overexpression

**Fig. 5:**
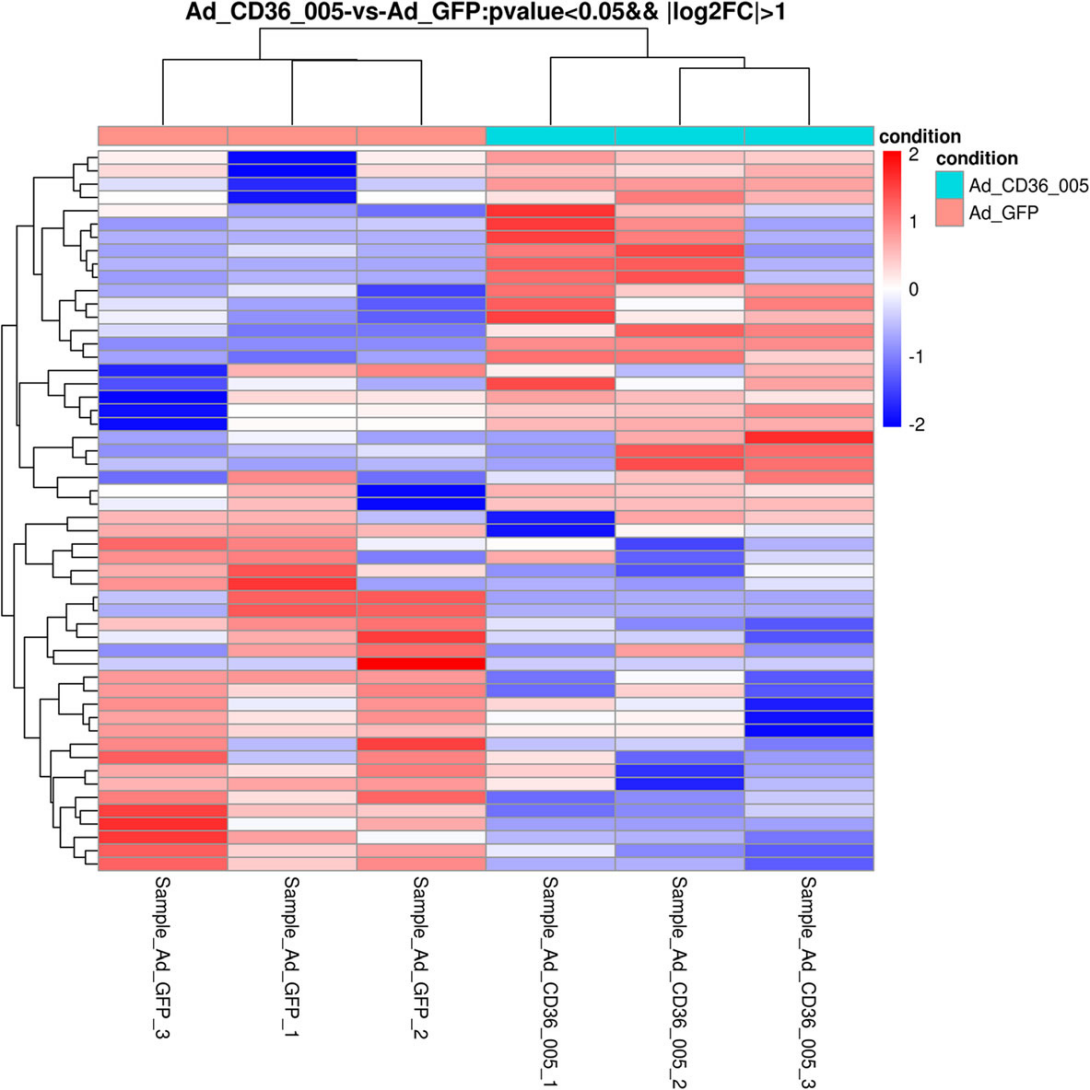
Hierarchical clustering heatmaps of differentially expressed mRNAs in the endometrial stromal cells after lncRNA CD36–005 overexpression

A total of 55 mRNAs were differentially expressed between the Ad-CD36–005 and Ad-GFP groups (absolute log_2_(fold change) > 1, *p* < 0.05) (Fig. 5). Moreover, 22 mRNAs were differentially expressed with absolute log_2_(fold change) > 2. Among the 55 differentially expressed mRNAs, 28 mRNAs were upregulated and 27 mRNAs were downregulated in the Ad-CD36–005 group.

### Validation of differentially expressed mRNAs

**Fig. 6:**
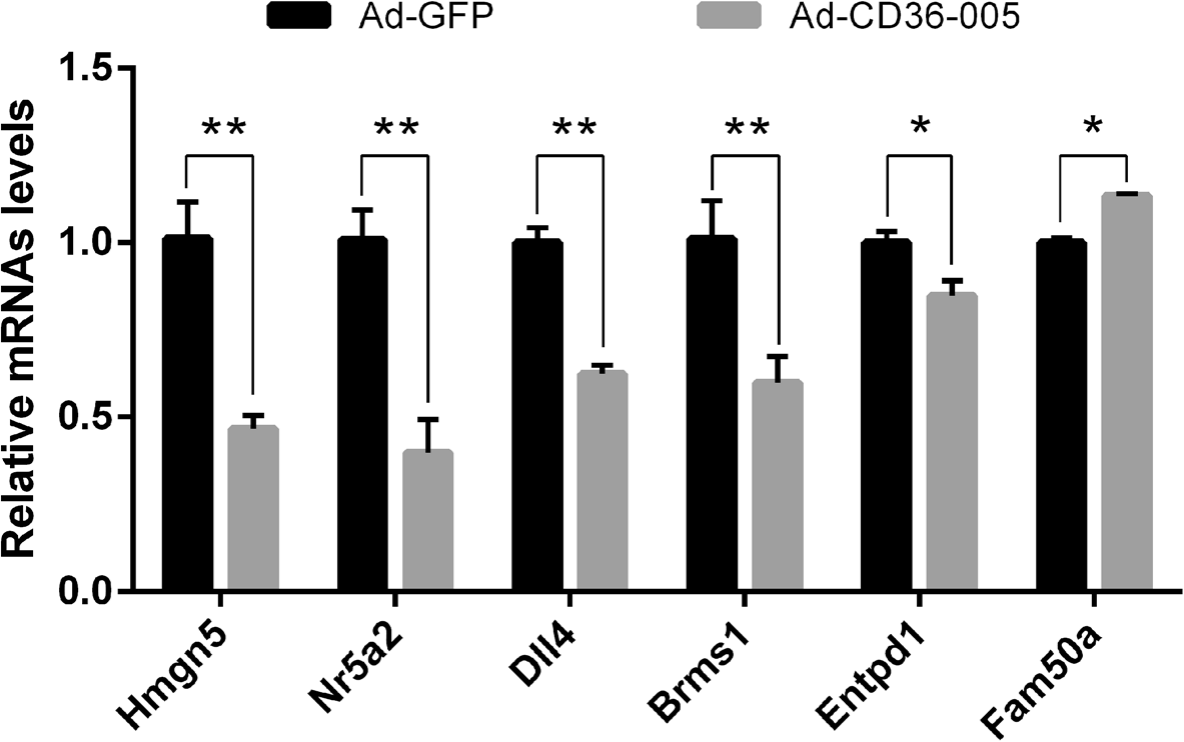
Relative expression of six selected mRNA was quantified by qRT-PCR. Compared with the Ad-GFP group, Hmgn5, Nr5a2, Dll4, Entpd1, and Brms1 displayed decreased expression, whereas Fam50a displayed an increased expression in the Ad-CD36–005 group (* *P* < 0.05; ** *P* < 0.01). Data are shown mean ± SEM

According to references and our interest, six mRNAs from the results of RNA-seq were selected for further validation by using qRT-PCR analysis. Compared with the Ad-GFP group, Hmgn5, Nr5a2, Dll4, Entpd1, and Brms1 displayed a decreased expression, and Fam50a displayed an increased expression in the Ad-CD36–005 group (p < 0.05) (Fig. 6). These results showed that the qRT-PCR results of expression levels of all six mRNAs confirmed were consistent with the RNA-seq data.

### Bioinformatics analysis

**Fig. 7:**
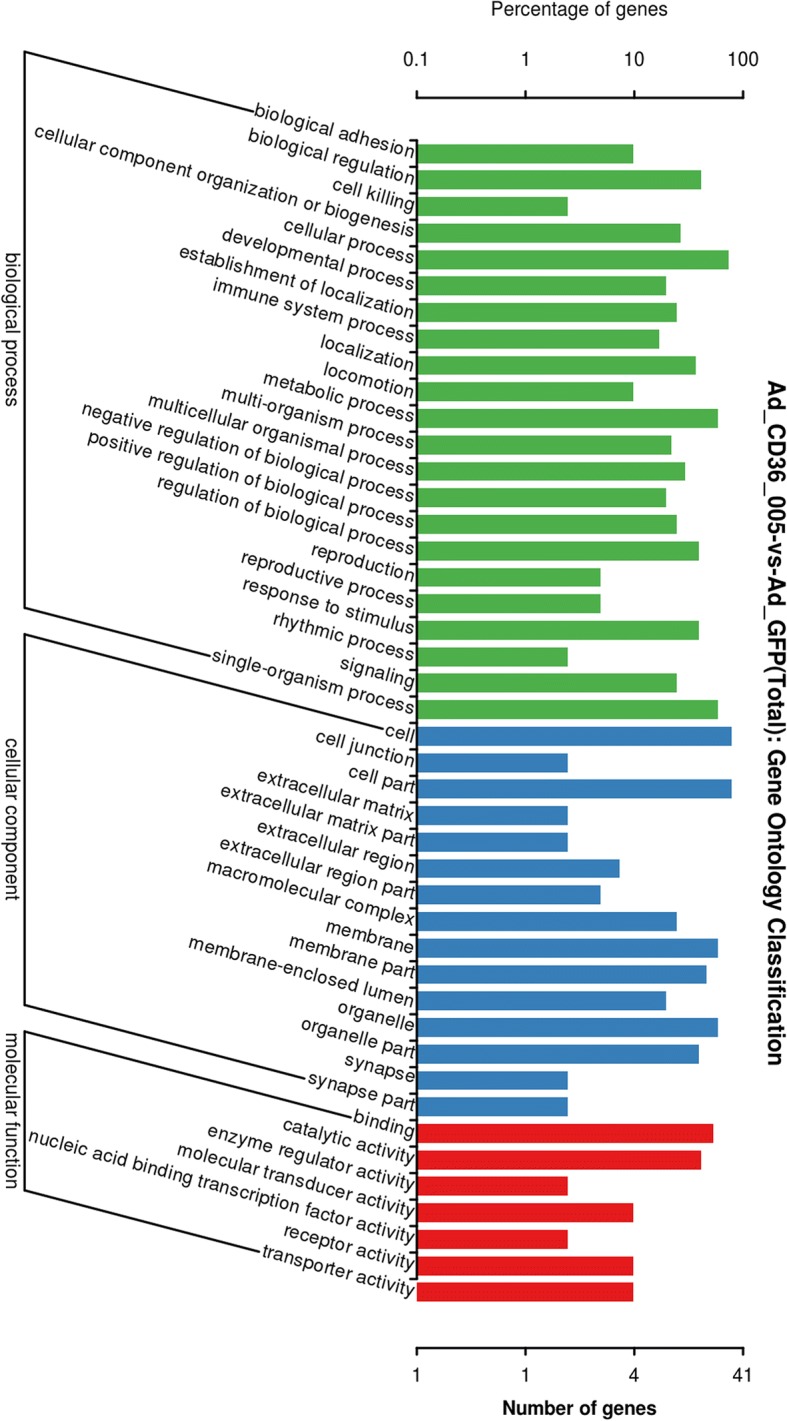
Gene Ontology (GO) classification of differentially expressed mRNAs. GO annotation showed that the differentially expressed mRNAs were associated with different biological processes

**Fig. 8:**
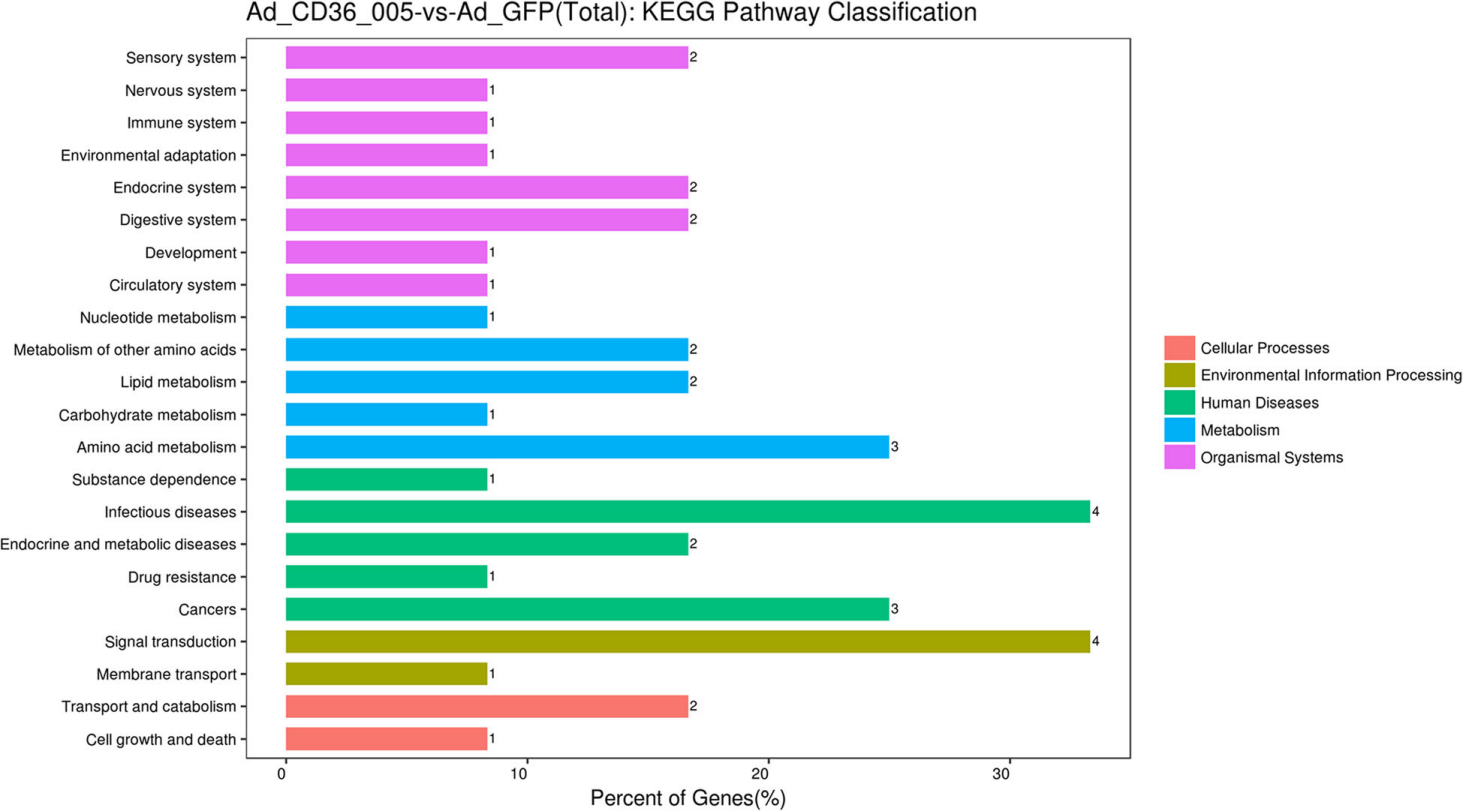
Kyoto Encyclopedia of Genes and Genomes (KEGG) pathway classification of differentially expressed mRNAs. KEGG pathway analysis showed that the differentially expressed mRNAs were involved in different signaling pathways

Results of the GO analyses categorized the differentially expressed mRNAs into different biological processes, such as biological adhesion, reproductive process, and metabolic process (Fig. 7). KEGG pathway analyses predicted that the differentially expressed mRNAs were involved in various biochemical pathways, including cell growth and death, transport and catabolism, signal transduction, lipid metabolism, and so on (Fig. 8).

## Discussion

Accumulating evidence shows that the endocrinal and metabolic disorders of PCOS have complex effects on the endometrium, leading to endometrial abnormalities [11, 14]. The endometrium is a steroid hormone-targeting tissue that undergoes cyclic secretion and proliferation dynamically in response to estrogen and progesterone produced by the ovaries [30]. Because most patients with PCOS are anovulatory or oligo ovulatory, the endometrium is continuously stimulated by unopposed estrogen in the absence of the regulatory effects of progesterone, which decrease the endometrial receptivity and promote the development of endometrial hyperplasia and even cancer in the long run [11]. In addition, patients with PCOS often have IR and obesity [6]. Insulin levels in the local endometrium affect the endometrial development and receptivity. IR can cause hyperglycemia, which further aggravates hyperandrogenism [14]. These are all high risk factors of endometrial cancer [11]. The pathogenesis of PCOS and its abnormal endometrial outcomes is a multifactorial biological process that involves a large number of genes and biological pathways, among which the role of lncRNAs has been studied in recent years [22, 31].

Owing to the abundance but low expression level of lncRNAs, they were initially considered to be transcriptional noise without any biological function [32]. With the rapid development of genomics and transcriptomics technology, researchers gradually found the regulatory role of lncRNAs in various human diseases [17]. In accumulating studies, only a few lncRNAs have been reported to be associated with both PCOS and various endometrial disorders. The expression of steroid receptor RNA activator (SRA) as well as lncRNA CTBP1-AS, a novel androgen receptor modulator, was significantly higher in peripheral blood leukocytes of women with PCOS. Meantime, it is known that women with PCOS show dysregulated hormone receptors expression, suggesting us a potential role of genes modulating hormone receptors in PCOS-associated endometrial disorders [33–35]. However, the functions and underlying mechanisms of these dysregulated remains unclear and need to be further studied.

The endometrial stromal cell is one of the in-vitro models of the endometrial tissue used for the study of the molecular mechanisms of endometrial diseases. Decidualization is a process of endometrial stromal cell proliferation and subsequent differentiation, during which stromal cells transform into specialized decidual cells [36, 37]. This process is essential for embryo implantation and successful pregnancy. Although many studies have focused on the molecular mechanisms of decidualization, known lncRNAs associated with decidualization were limited. HK2P1 is a lncRNA found to be decreased in the decidua of severe preeclampsia patients. In vitro results show that downregulated HK2P1 inhibited human endometrial stromal cell (HESC) proliferation and differentiation by regulating miR-6887-3p and its target gene HK2 expression as a ceRNA [38]. The expression of another lncRNA, LINC00473, is highly induced in HESCs after decidual stimulus [39]. These studies prove a crucial role of lncRNA in stromal cell decidualization and suggest that lncRNAs participate in some endometrial diseases partly through affecting stromal cells, of which some may be caused by PCOS.

In the present study, CD36–005 was upregulated in the uteri of PCOS rat model and could promote the proliferation of stromal cells. However, the molecular mechanisms remain to be characterized. Thus, we conducted RNA-seq and found 55 known mRNAs in the primary endometrial stromal cells differentially expressed between the Ad-CD36–005 and Ad-GFP group with a threshold of *p* < 0.05 and |log_2_(Fold-Change)| > 1. We chose six mRNAs (Hmgn5, Nr5a2, Dll4, Entpd1, Fam50a, and Brms1) for qRT-PCR validation, and the results were consistent with the RNA-seq data. In previous studies, Qiao performed microarray analysis on the endometrial biopsies of women with PCOS during the implantation window and found down-regulated genes were associated with endometrial receptivity [40]. Similarly, Bellver performed microarray hybridization and identified an aberrant endometrial transcriptome in obese women with PCOS during the implantation window [41]. Among our RNA-seq result, several genes have been reported to be involved in endometrial disorders, but little is known whether these endometrial disorders are related to PCOS. Hmgn5, also known as Nsbp1, regulates uterine decidualization as a downstream gene of Hoxa10 in a differentiation-specific manner [42]. Hmgn5 was also found to be hypomethylated in mouse uteri when exposed to diethylstilbestrol or fenistein neonatally [43]. Nr5a2, also known as Lrh-1, was known as a key transcriptional factor of multiple steroidogenic genes in vitro [44]. In Wang’s study, Nr5a2 promotes aromatase expression in primary rat granulosa cells, indicating its potential involvement in PCOS while the ovaries of women with PCOS have abnormal steroidogenesis and folliculogenesis [45, 46]. Consistent with above report, Kevin elucidated in his review that a higher level of Lrh-1 could activate estradiol production in the endometrium of women with endometrial cancer [47]. Lrh-1 is also essential for a successful pregnancy as its indispensible roles in the luteal function, decidualization, and placental formation [48]. Combined with our result, we could speculate that the dysregulated expression of Nr5a2 may be associated with PCOS-associated endometrial disorders. Dll4, a gene involved in the delta-notch pathways, participates in the decidualization failure of stromal cells from women with endometriosis [49]. The promiscuous expression of Dll4 impaired decidual angiogenesis, and coordinated with disrupted decidual cellular proliferation and apoptosis, could be one of the causes of early miscarriages [50]. No relevant studies focus on the relationship of Entpd1, Fam50a, and Brms1 to endometrial disorders, and further studies are needed.

GO and KEGG analyses were performed to identify the potential functions and pathways of the 55 target genes of CD36–005. GO analyses revealed that these differentially expressed genes were involved in various categories, such as nitric oxide biosynthetic process and positive regulation of gene expression including toll-like receptor 7/9 (TLR-7/9) and interleukin-6/8 (IL-6/8). The nitric oxide (NO)/ nitric oxide synthase (NOS) system was presumed to locally regulate the endometrial functions, including stromal cells decidualization [51]. Nos2, a synthase involved in NO production, was upregulated by CD36–005 overexpression and predicted to be involved in the nitric oxide mediated signal transduction, nitric-oxide synthase activity, and nitric-oxide synthase binding. Nos2 was also found to participate in the inflammatory process of PCOS and some endometrial abnormalities [52, 53]. Thus, we can speculate that Nos2 may play a role in the pathogenesis of endometrial abnormalities of PCOS, however, there is no direct evidence and further studies are needed. TLR-7/9 and IL-6/8 were shown to be related to endometrial disorders. The expression of TLR-9 was increased with reduced DNA methylation in spontaneous preterm labor [54]. Higher TLR-9 transcriptional activity may be a protective factor for endometrial cancer risk [55]. According to Gu, the expression of TLR9 in cumulus cells was influenced significantly by PCOS, which may further lower the embryo quality and decrease the fertility rate of women with PCOS. These results suggest us the dysregulated expression of TLR9 may be potentially involved in the pathogenesis of PCOS and its adverse endometrial outcomes. TRL-7 is crucial in the establishment and maintenance of pregnancy in sheep [56]. IL-8 participates in the pathogenesis of endometriosis by regulating ectopic endometrial cell proliferation, invasion, and adhesion [57]. Levels of IL-6 in the mid-secretory-phase endometrium are lower in women with previous recurrent miscarriage [58]. Additionally, the aberrant IL-6 and IL-8 in endometrial stromal fibroblasts from women with PCOS was thought be related to the altered endometrial immune profile and imbalanced leukocyte migration, both of which contribute to a sub-optimal implantation of women with PCOS [59]. Thus, the regulation of these biological processes may be one of the complex mechanisms of some endometrial abnormalities, of which some might be induced by PCOS.

KEGG analyses showed that these differentially expressed genes were associated with diverse signaling pathways, including cell growth and death, transport and catabolism, signal transduction, lipid metabolism, endocrine and metabolic diseases, and cancers. Among these pathways, lipid metabolism was a biological process closely related to PCOS and its endometrial abnormalities. In previous studies, 70% of women with PCOS were found to have dyslipidemia, which is also a risk factor for cardiovascular disease and endometrial cancer [60]. Patients with PCOS commonly suffer from increased body mass index, total cholesterol, triglyceride, low-density lipoprotein-cholesterol, and decreased high-density lipoprotein-cholesterol [61]. In Chekir’s study, patients with PCOS have an increased uterine arterial pulsatility index and reduced endometrial thickness during the luteal phase, which may cause reproductive failure. The endometrial dysfunction induced by impaired uterine perfusion was thought to be correlated with dyslipidemia [61]. Nieman regarded endometrial cancer as the most relevant cancer with obesity. Adipocytes secrete adipokines and mediate the transition of androgens to estrogen, which promotes the development of endometrial cancer [62]. Mogat1 is a gene upregulated by CD36–005 overexpression and predicted to be involved in lipid metabolism. Hence, the differential upregulation of Mogat1 may participate in the pathogenesis of PCOS and its endometrial abnormalities through lipid metabolism. Above all, these results suggest a potential mechanism through which the dysregulated mRNAs involved in different categories and biological pathways contribute together to the regulation of stromal cells and the pathogenesis of endometrial abnormalities, some of which may be caused by PCOS.

## Conclusion

We identified a list of 55 potential target mRNAs of CD36–005 in the primary endometrial stromal cells by RNA-seq and confirmed the relative expression levels of Hmgn5, Nr5a2, Dll4, Entpd1, Fam50a, and Brms1 using qRT-PCR. We used GO and KEGG pathway analyses, and predicted the various biochemical pathways that regulate the proliferation and decidualization of stromal cells and the pathogenesis of some endometrial abnormalities, in which these differentially expressed mRNAs may be involved in. Our results partly explained the mechanisms by which CD36–005 regulate the etiology and pathophysiology of PCOS in the uterus of rat with letrozole-induced PCOS, and laid a foundation for further studies on the molecular function and mechanism of CD36–005 in stromal cells. Further detailed studies are needed to clarify the underlying mechanisms of CD36–005 on the regulation of stromal cells and to investigate if these alterations could serve as biomarkers for the prediction of some endometrial diseases induced by PCOS.

## Additional files

Additional file 1: Table S1. The raw data for Figure 3. (XLSX 9 kb)

Additional file 2: Table S2. The raw data for Figure 4. (XLSX 9 kb)

## Abbreviations

CD36–005: lncRNA CD36–005
E_2_: 17-beta estradiol
GO: Gene Ontology
HESC: human endometrial stromal cell
IL: interleukin
IR: insulin resistance
KEGG: Kyoto Encyclopedia of Genes and Genomes
LncRNA: long non-coding RNA
MOI: multiplicity of infection
NO: nitric oxide
NOS: nitric oxide synthase
PCOS: polycystic ovary syndrome
RNA-seq: RNA sequencing
SRA: steroid receptor RNA activator
TLR: toll-like receptor
